# Comprehensive Laboratory Diagnostic Workup for Patients with Suspected Intraocular Lymphoma including Flow Cytometry, Molecular Genetics and Cytopathology

**DOI:** 10.3390/curroncol29020065

**Published:** 2022-01-31

**Authors:** Evgenii Shumilov, Paolo Mazzeo, Martin S. Zinkernagel, Myriam Legros, Naomi Porret, Lorenz Romagna, Detlef Haase, Georg Lenz, Urban Novak, Yara Banz, Thomas Pabst, Ulrike Bacher

**Affiliations:** 1 Department of Hematology and Central Hematology Laboratory, Inselspital, Bern University Hospital, University of Bern, 3012 Bern, Switzerland; Evgenii.Shumilov@ukmuenster.de (E.S.); Myriam.Legros@insel.ch (M.L.); NaomiAzur.Porret@insel.ch (N.P.); 2 Department of Medicine A, Haematology, Oncology, and Pneumology, University Hospital Münster, 48149 Münster, Germany; Georg.Lenz@ukmuenster.de; 3 Department of Hematology and Medical Oncology, University Medicine Göttingen (UMG), 37075 Göttingen, Germany; paolo.Mazzeo@med.uni-goettingen.de (P.M.); detlef.haase@med.uni-goettingen.de (D.H.); 4 Department of Ophthalmology, Inselspital, Bern University Hospital, University of Bern, 3012 Bern, Switzerland; martin.zinkernagel@insel.ch; 5 Department of Medical Oncology, Inselspital, Bern University Hospital, 3012 Bern, Switzerland; lorenz.romagna@insel.ch (L.R.); Urban.Novak@insel.ch (U.N.); Thomas.Pabst@insel.ch (T.P.); 6 Institute of Pathology, University of Bern, 3012 Bern, Switzerland; yara.banz@pathology.unibe.ch

**Keywords:** intraocular lymphoma, vitreous body, flow cytometry, molecular genetics, *MYD88*, cytopathology

## Abstract

Background: Intraocular lymphoma (IOL) presents a real challenge in daily diagnostics. Cyto- and/or histopathology of vitreous body represent the diagnostic cornerstones. Yet, false negative results remain common. Therefore, we analyzed the diagnostic significance of flow cytometry (FC) within the workup algorithm of IOL and compared its sensitivity with the results obtained from routine cytopathology and molecular genetics; Methods: Seven patients undergoing vitrectomy due to suspected IOL were investigated by FC and parallel cytopathology and, if available, digital droplet PCR (ddPCR) for *MYD88* L265P; Results: Four out of seven patients were finally diagnosed with IOL. Among the IOL patients, cytopathology confirmed the presence of lymphoma cells in only two cases. In contrast, FC was positive for IOL in all four cases, and FC additionally confirmed the lack of IOL in the remaining patients. In IOL patients diagnosed by FC and with available ddPCR, the diagnosis of IOL was confirmed by the presence of the *MYD88* L265P mutation in all three patients; Conclusions: The combination with FC was superior to cytopathology alone in the diagnostic work-up of IOL, and it showed an excellent correlation with ddPCR results. A comprehensive diagnostic panel consisting of cytopathology, FC and molecular genetics should be considered for the work-up of suspected IOL.

## 1. Introduction

Intraocular lymphoma (IOL) is a rare group of lymphoid neoplasms accounting for up to 2% of ocular malignancies and extranodal lymphomas as well as 4–6% of primary central nervous system (CNS) tumors [1,2,3]. IOL includes two forms, namely, primary IOL (PIOL) arising in the central nervous system (CNS) and representing a subset of primary (CNS) lymphoma (PCNSL) [4], and secondary IOS (SIOL) which derives from a systemic malignant lymphoma [5,6]. Commonly, IOL arise in the vitreous body or in the retina referred accordingly to as vitreoretinal lymphoma (VRL) [7]. Rarely, IOL may occur in the uveal tract (iris, ciliary body, and choroid) which is referred to as uveal lymphomas presented mostly by indolent extranodal marginal zone lymphoma [8]. Mostly, VRL comprises aggressive diffuse large B-cell lymphoma (DLBCL; ≥95%) although intraocular T-cell lymphomas have also rarely been reported [9,10,11,12].

The diagnosis of IOL frequently represents a challenge and can result in a relevant treatment delay. If untreated, 50% of PIOL cases experience lymphoma progression towards additional non-ocular CNS compartments [5]. Clinically, PIOL is a masquerader of common eye disorders, delaying diagnosis on average for more than one year from onset of symptoms until ultimate diagnosis [4,13]. During this time, patients may develop uveitis and may be insufficiently treated with corticosteroid monotherapy. Given the lympholytic effect of steroids, B-cell lymphoma may remain undetectable in the vitreous body, resulting in further diagnostic delay.

Vitreous biopsy with subsequent cytology and histopathology of the vitreous body represents the gold standard for the diagnosis of IOL. Acknowledging the value of histological diagnosis, false negative results are common in IOL cases. Indeed, factors such as the fragility of lymphoma cells, low cell counts within the vitreous sample, preceding steroid treatment, and presence of other cells or debris can hamper the identification of lymphoma cells [14]. Therefore, molecular genetics may significantly contribute to the diagnosis of PIOL, since up to 80% of PVRL cases are positive for the *MYD88* L265P mutation detected by either polymerase-chain reaction (PCR) or droplet digital PCR (ddPCR) [15,16,17]. Although IGH rearrangement can also be tested in PIOL, it requires more cells and is associated with a higher rate of false-negative results compared to *MYD88* L265P testing [15]. Given that IOL can also be associated with CNS presentations as a part of PCNSL or the progression of systemic lymphoma, magnetic resonance imaging (MRI) should be part of the diagnostic work-up if IOL is considered. Although flow cytometry is more sensitive for the identification of a clonal B-cell population, its integration within the diagnostic workup of IOL remains limited to a handful of cases only [18].

In particular, the limited availability of cell numbers in the vitreous fluid has so far impeded the use of flow cytometry for IOL diagnostics. In this single-center study, we report the successful implementation of flow cytometry in patients with suspected IOL in combination with cytopathology and molecular genetics. We also propose a comprehensive diagnostic algorithm of IOL.

## 2. Materials and Methods

### 2.1. Patients

Vitreous samples from seven patients admitted to the University Hospital of Bern, Switzerland, between June 2017 and June 2020 undergoing vitrectomy due to suspicion of IOL or uveitis of unknown origin were investigated by flow cytometry in addition to routine parallel histopathologic (cytopathology) and, if available, molecular genetic analysis at the same institution. Patients were included in the analysis based on the availability of flow cytometry from vitreal sampling. Informed consent was obtained from all individual participants included in the study.

### 2.2. Cytopathology

Cytology specimens derived from the central nervous system and, in particular, the intraocular compartment typically present as small volume (paucicellular) specimens. Cytological analysis of lymphoid cells in the setting of suspected IOL is often hampered by the low cell counts and the work-up of the specimen necessitating spin-down steps, which inherently alter the cytomorphological appearance of the fragile (both neoplastic and non-neoplastic) lymphoid cells. If, however, sufficient material is available and the cell count is high enough, the preparation of a cell block, which could allow for immunohistochemical stainings, may potentially increase diagnostic sensitivity [19]. In the current cases, the cell count was too low to prepare cell block specimens. Therefore, fresh specimens (containing 1.5 mL each) were centrifuged for 1 min at 1700 rpm and cytospin preparations were obtained. Specimens were fixed in 100% alcohol and stained with Papanicolaou using the Tissue-Tek Prisma stainer (Sakura Finetek, Torrance, CA, USA). If sufficient material was available, samples were further split for flow cytometry and molecular pathology (see below).

### 2.3. Flow Cytometry

Due to the reduced stability of vitreous material, samples had to be transferred to the flow cytometry lab immediately following the biopsy procedure to undergo further processing. Considering the limited pre-analytic amount of sample material, processing was omitted in favor of direct analysis of material on the flow cytometer. In the first step, samples were analyzed using the lymphocyte screening tube (Cytognos S.L., Salamanca, Spain) established by the EuroFlow Consortium [20]. The combination of B-cell (CD19, CD20), T-cell (CD3, CD4, CD8, CD5), NK (CD56), and clonality markers (light chains kappa/lambda) allows for both the detection of lymphocyte subsets and malignant B-cells. If the screening showed an increase in B cells numbers and/or an aberrant expression pattern (including clonality detection due to light chain expression), an expanded additional lymphoma panel containing the markers CD79b, CD23, CD22, CD10, CD200, FMC7 and LAIR1 (Becton Dickinson Biosciences, BD, San Jose, CA, USA) was used if sufficient material was available. Samples were analyzed on a FACS Canto II and analyzed using Diva software (Becton Dickinson Biosciences, BD, San Jose, CA, USA).

### 2.4. Molecular Genetics

Genomic DNA was extracted from very small samples using the QIAamp DNA mini kit (Qiagen, Hombrechtikon, Switzerland). Droplet digital PCR (ddPCR) was carried out using the Bio-Rad assay for *MYD88* L265P (Bio-Rad, Cressier, Switzerland) with HaeIII as digestion enzyme (New England Biolabs, Ipswich, MA, USA), according to the manufacturer’s instructions. After droplet generation on the AutoDG automated droplet generator (Bio-Rad, Cressier, Switzerland) and PCR, the readout was performed on the QX200 Droplet Reader (Bio-Rad, Cressier, Switzerland) with the two-color detection system set to FAM and HEX. IGH rearrangements were analyzed using the IdentiCloneTM IGH Clonality Assay Kit (IVD Kit according to BIOMED2; Invivoscribe Inc., San Diego, CA, USA).

### 2.5. Imaging

The MRI protocol of the brain and orbits included axial and coronal T2-weighted imaging and T1 without and with contrast, as well as fat-suppression T1-weighted imaging with contrast assistance.

## 3. Results

### 3.1. Patient Characteristics

Vitreal samples from seven patients were investigated by flow cytometry due to suspected IOL. The clinical, instrumental and laboratory findings of these patients are presented in Table 1. Following comprehensive diagnostics, four out of seven patients with vitrectomy were, finally, diagnosed with IOL. In the fifth patient with progression of systemic DLBCL in the CNS, IOL was excluded. The remaining two patients had no findings of IOL or systemic lymphoma. Among the four patients with IOL, three patients were older than 60 years (two males, two females). At diagnosis of IOL, two patients presented with a progressive loss of visual acuity and two had symptoms similar to uveitis. All four patients had PIOL—two as a clinical manifestation of PCNSL and the remaining two as isolated ocular lymphoma.

### 3.2. Diagnostic Power of Cytopathology, Flow Cytometry, and ddPCR/PCR in Vitreous Samples

#### 3.2.1. Patients with Intraocular Lymphoma

##### Cytopathology

The results of cytopathology are presented in Table 1 and Table 2. Out of four patients with the final diagnosis of IOL, cytopathology confirmed the presence of lymphoma cells in two cases (#2, 3) only.

##### Flow Cytometry

Flow cytometry detected a monoclonal B-cell population in four patients (#1–4) and was negative in two patients (#6–7). In the remaining case (#5), acellular punctate was present (Table 1 and Table 2). In all four lymphoma samples, B-lymphocytes (CD19+/CD20+) represented the majority of cells of the vitreous body material ranging from 30% to 84% of all evaluable cell events. There was no aberrant antigen expression with regard to CD5 or CD10 in IOL cases. Three of these four patients showed surface light-chain restriction for kappa and in a single case for lambda. Immunophenotypes are shown in more detail in Table 2. A clear assignment to a specific lymphoma subtype was not possible in the respective four cases, partially because the antibody panel or number of tubes was limited due to the small sample size and low cell numbers. 

##### ddPCR/PCR

ddPCR was performed in three of the four patients with a final diagnosis of IOL. These three cases (#1, 2, 4) were positive for *MYD88 p*.L265P (Table 1). In one of these cases (#2), an additional clonal IGH-gene rearrangement (by PCR) was identified. In two out of three cases (#1, 4) with evidence of a *MYD88* mutation, cytopathology was not conclusive.

#### 3.2.2. Patients without Evidence of Intraocular Lymphoma

##### Cytopathology

Three patients (#5–7) showed no evidence of IOL by any of the laboratory methods listed above or by MR imaging. Cytopathology was performed in only one of these patients who showed progression of DLBCL with CNS involvement, and this patient showed no evidence of lymphoma by cytopathology.

##### Flow Cytometry

Vitreous samples from all three cases without subsequent evidence of IOL (#5–7) underwent investigation by flow cytometry. None of these samples comprised B-lymphocytes in the vitreous bodies. Notably, the patient with previously known DLBCL and progression within the CNS (#5) showed no evidence of cells in the vitreous sample. Of the remaining two cases with uveitis, one patient (#7) had predominantly T-lymphocytes with a physiologic phenotype in the vitreous body sample. According to the results of flow cytometry in these three patients, there was no indication for ddPCR/PCR due to the lack of vitreous B-lymphocytes.

##### Correlation of Cytopathology and Flow Cytometry in Vitreous Samples

The correlation of the results of cytopathology and flow cytometry among all seven investigated patients is presented in Table 2. Of the five patients with available cytopathology and flow cytometry, results were concordant in three cases (60%; #2, 3, 5) including two patients with IOL and one with exclusion of IOL following progression of DLBCL in the CNS. However, it should be emphasized that two patients (#1, 4) showed no evidence of IOL by cytopathology but a clear presence of IOL by flow cytometry. Notably, vitreous samples from both patients were positive for the *MYD88 p*.L265P mutation by ddPCR, with a mutation load of approximately 50% confirming the results of flow cytometry. Thus, in two cases of our cohort, the combined use of flow cytometry and molecular genetics was more efficient to detect IOL compared to cytopathology only.

##### Diagnostic Value of Imaging in Patients of the Study

MRI of the brain was performed in all five lymphoma patients (4 IOL cases, 1 case with secondary CNS lymphoma following progress of DLBCL) (Table 1). Accordingly, two patients with IOL (#1, 3) had no evidence of CNS involvement by MRI, whereas three other patients showed cerebral lesions. Of the latter, two cases (#2, 4) had evidence of IOL and the third one (#5) lacked signs of IOL by cytopathology and flow cytometry. Based on MRI results and findings in vitreous samples, two patients were assigned to isolated IOL, two other patients to IOL within PCNSL, and the remaining patient to secondary CNS lymphoma without intraocular involvement following progression of DLBCL.

##### Combination of Cytopathology, Flow Cytometry, Molecular Genetics and Imaging for Diagnostics of IOL

Combining all four methods, isolated IOL with B-cell origin could be clearly diagnosed in 4 out of 7 patients.

## 4. Discussion

IOL represents a challenging diagnosis requiring a thorough diagnostic workup as part of a multidisciplinary approach. Although cytopathology plays a crucial role in diagnosing IOL, additional methods including molecular genetics or flow cytometry have increasingly been applied in these settings [7,21]. We propose a comprehensive diagnostic algorithm including cytopathology, ddPCR, flow cytometry and imaging. By applying such a conclusive panel combining these methods in seven patients selected for possible lymphoma, we were able to diagnose IOL in 4 cases.

Based on cytopathology alone, the diagnosis of IOL would have been missed in half of the patients. In contrast, flow cytometry allowed for the diagnosis of IOL in all patients with intraocular involvement. So far, only single reports on the application of flow cytometry in vitreous specimens from IOL patients are available [18,22,23]. In line with our analysis, Davis and colleagues reported a high sensitivity of flow cytometry in patients with vitreous cellular infiltration resulting in IOL confirmation in seven of ten patients (70%). Yet, cytopathology was only able to detect three of the ten patients (30%) [18]. Recently, Cantu and colleagues reported on the largest retrospective analysis of vitreous samples with concomitant cytopathology and flow cytometry. Of 73 specimens collected for suspected lymphoma, 15 (21%) were subsequently positive or suspicious for IOL by cytopathology. Accordingly, nine of these 15 cases (60%) had evidence of IOL by flow cytometry whereas the remaining six cases lacked confirmatory flow cytometry due to insufficient amount of available vitreous cells. Of note, none of the samples without large lymphocytes by cytopathology showed evidence of lymphocyte abnormalities by flow cytometry. Thus, the authors postulated that inconspicuous cytology examination in vitreous specimens should preclude the use of flow cytometry [22]. However, based on such an approach, two out of four IOL patients from our cohort would not meet the diagnostic criteria for IOL.

It should be mentioned that flow cytometry can detect antigen patterns that may be characteristic for certain lymphoma subtypes, but a clear delineation of a distinct lymphoma entity is possible only for few entities that play a minor or no role for IOL (e.g., hairy cell leukemia or chronic lymphatic leukemia, CLL). Therefore, flow cytometry will be rather helpful for identifying B-cell lymphoma in the vitreal body but, for the delineation of the exact subtype, cytopathology will be more important.

Acknowledging technical limits of cytopathology and flow cytometry in vitreous samples, molecular genetics represents an additional tool in the diagnostic workup of IOL. Indeed, in all our IOL cases diagnosed by flow cytometry and with available ddPCR, the diagnosis of IOL was confirmed by the presence of the *MYD88* L265P mutation. Importantly, one of these IOL cases with mutated *MYD88* L265P would have been missed by cytopathology alone. Thus, if the *MYD88* allele-specific PCR is positive in the context of CD19+/CD20+ cells in vitreous sample, a diagnosis of IOL can be confirmed [15].

In order to distinguish different forms of IOL (e.g., isolated PIOL, PCNSL, or SIOL), imaging, in particular MRI of the brain, plays a crucial role in the identification of additional IOL CNS manifestations. Moreover, peripheral imaging (e.g., CT or PET-CT) is also crucial to exclude secondary involvement of the eye. The results of both, in turn, have a direct impact on the choice of subsequent treatment strategies. For the overall diagnostic approach, cerebrospinal fluid (CSF) analysis can be recommended as well to exclude occult CNS manifestations within a PCNSL or SIOL helping to differentiate it from PIOL.

**Figure 1:**
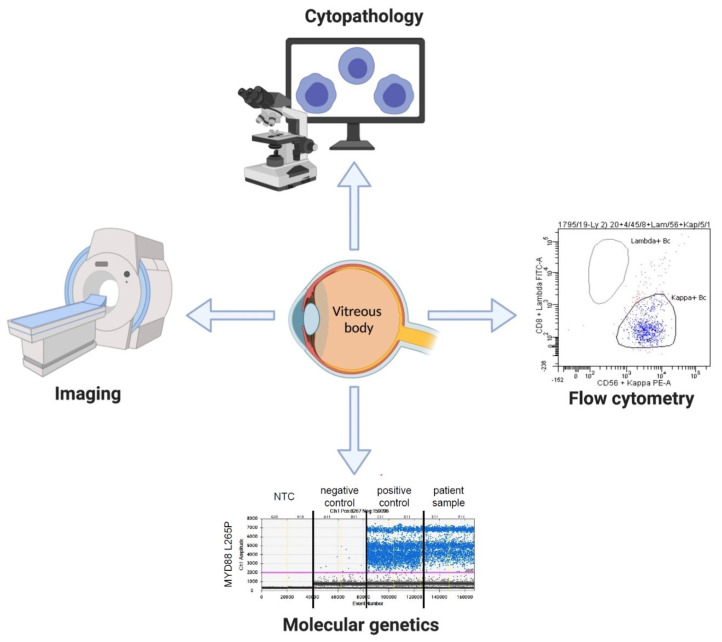
Diagnostic panel for intraocular lymphoma.

Based on our experience in the diagnostic work-up of IOL, we propose a comprehensive multidisciplinary diagnostic approach for intraocular lymphoma using cytopathology, flow cytometry, molecular genetics and imaging (Figure 1). The analyses performed on vitreal body should be combined with other diagnostic techniques and methods that may be necessary in the clinical context (e.g., detection of paraproteinemia in *MYD88*-mutated cases).

In parallel, comprehensive clinical care of IOL patients with the involvement of ophthalmologists, neurologists and hemato-oncologists should be considered for optimal diagnostic and treatment decisions. Acknowledging the diagnostic power and limitations of each method, we believe that such a panel in combination with a thorough clinical assessment should allow for a correct and timely diagnosis IOL with a high sensitivity. Given the low frequency of IOL and challenges associated with its diagnosis, multidisciplinary boards and multicenter collaborations are crucial to further improve the diagnostic approaches in patients with suspected IOL.

## 5. Conclusions

Intraocular lymphoma is a challenge in daily diagnostics. A comprehensive diagnostic panel consisting of cytopathology, flow cytometry, molecular genetics and imaging should be considered for the workup of suspected intraocular lymphoma. A multidisciplinary approach plays a crucial role for optimal diagnostic and treatment decisions.

## Figures and Tables

**Table 1:** Overview on seven patients with confirmed or excluded intraocular lymphoma. Yrs, years; DLBCL, diffuse large B-cell lymphoma; R-CHOP, rituximab, cyclophosphamide, daunorubicin, vincristine, prednisone; PR, partial remission; CSF: cerebrospinal fluid; MRI, magnetic resonance imaging; CNS, central nervous system; MTX, methotrexate; FU, follow-up; mo., month(s); CT, computed tomography; BM, bone marrow; neg, negative; ddPCR, digital droplet polymerase chain reaction; IGH, immunoglobulin heavy locus; MATRIX, methotrexate, cytarabine, thiotepa, rituximab; HDCT/ASCT, high-dose chemotherapy/autologous stem cell transplantation; PET, positron emission tomography.

Patient No	Age/ Gender	History of Lymphoma/Other Conditions	Clinical, Instrumental and Histopathology Findings Accompanying Vitrectomy	Investigation of Vitreous Body	Final Diagnosis	Treatment/Clinical Course
***Lymphoma patients***						
**#1**	88 yrs, female	Behçet’s disease	granulomatous uveitis; *MRI*: no signs of intracerebral tumor; *CT*: no lymphadenopathy; pathology (BM): no lymphoma;	*flow cytometry*: CD5 neg. monoclonal B-cell population (92% of all cells); *cytopathology*: no evidence of lymphoma cells; *ddPCR*: mutated *MYD88* (*p*.L265P) (50.5%)	isolated intraocular B-cell lymphoma	corticosteroid pulse therapy; last FU (1 mo): death from sepsis
**#2**	67 yrs, male	No	bilateral visual impairment; *MRI*: perivascular lesions periventricular; *CT*: no lymphadenopathy; pathology (BM): no lymphoma	*flow cytometry*: CD5/CD10 neg. monoclonal B-cell population (51% of all cells); *cytopathology*: infiltrates of CD5/CD10 neg. B-cell neoplasm; *PCR*: clonal IGH-gene rearrangement *ddPCR*: mutated *MYD88* (*p*.L265P) (49%)	primary B-cell CNS lymphoma with intra-ocular lymphoma	MATRIX x2 with PR; last FU (5 mo): death from sepsis in aplasia
**#3**	63 yrs, female	No	intermediate uveitis bilaterally; *MRI*: no signs of intracerebral tumor; *PET/CT*: no lymphadenopathy; *CSF*: inconspicuous	*flow cytometry*: CD5/CD10 neg. monoclonal B-cell population (33% of all cells); *cytopathology*: single atypical blastic lymphoid cells; laboratory chemistry: increased IL-10/IL ratio (34.2)	isolated B-cell intraocular lymphoma	radiotherapy of both eyes; last FU (7 mo): alive (in remission)
**#4**	53 yrs, male	epilepsy for years	progressive bilateral loss of vision; *MRI*: confluent bilateral medullary lesions; *CT*: no lymphadenopathy; *CSF*: inconspicuous	*flow cytometry*: CD5 neg. monoclonal B-cell population (45% of all cells); *cytopathology*: no evidence of lymphoma cells; *ddPCR*: mutated *MYD88* (*p*.L265P) pathology (brain): DLBCL, mutated *MYD88* (*p*.L265P)	primary CNS lymphoma (DLBCL) with intraocular lymphoma	MATRIX x4 followed by front-line HDCT/ASCT; last FU (13 mo): alive (in remission)
**#5**	80 yrs, male	DLBCL, IVb followed by R-CHOP x6 with PR	progressive loss of vision, nystagmus, headache, ataxia, oculomotor palsy both-sided; ophthalmological consultation: suspected intraocular lymphoma; *CSF*: suspicious lymphocytes; *MRI*: cerebral edema, lesions around fourth ventricle and in pons	*flow cytometry*: acellular punctate *cytopathology*: numerous macrophages and reactive T lymphocytes, no lymphoma cells	secondary CNS lymphoma following progress of DLBCL; no evidence of intraocular lymphoma	MTX x2 with stable disease; last FU (4 mo): death, non-cancer cause
***Non-lymphoma patients***						
**#6**	58 yrs, female	sarcoidosis-like disease with chronic alveolitis, arthritis and keratouveitis	dyspnea, decreased visual acuity in right eye; *PET/CT*: inflammatory bilateral pulmonary changes, no lymphadenopathy; *histopathology* (lung): unclassifiable interstitial pneumonia	*flow cytometry*: relative increase in NK cells, lack of B cells, T-cells 2% of all cells	no findings of intraocular lymphoma	long-term steroids; last FU (24 mo): alive, progressive pulmonary hypertension
**#7**	65 yrs, female	prurigo subacute; unspecific medullary lesions in MRI	recurrent bilateral uveitis with no response on MTX/steroids; *CT*: no lymphadenopathy	*flow cytometry*: relative increase in T-cells with increased CD4/CD8 ratio, no B-cells	no findings of intraocular lymphoma	last FU (4 mo): alive

**Table 2:** Detailed information on the results of flow cytometry and cytopathology of vitreous body in patients of this study. FC, flow cytometry; CP, cytopathology; CNS, central nervous system; DLBCL, diffuse large B-cell lymphoma; i.a, among others; NA, not available.

Patient No	Flow Cytometry (FC)	Cytopathology (CP)	Interpretation of Results	Final Diagnosis
***Lymphoma patients***				
**#1**	lymphocytes 92%, B-cells 84%, T-cells 3%, NK-cells 1% (of all evaluable cell events); B-cells: CD19+, CD20+, CD5-, CD10-, CD38+, CD79b+, CD81+, CD95+, CD200+, FMC7+, HLA-DR-, skappa+	*macroscopic*: colorless, clear liquid; *microscopic*: few normal lymphocytes and macrophages	discordance: lymphoma evidence by FC; lack of lymphoma evidence by CP	isolated intraocular B-cell lymphoma
**#2**	lymphocytes 87%, B-cells 51%, T-cells 12%, NK-cells 12%; B-cells: CD19+, CD20+, CD5-, CD10-, CD11c+, CD23+, CD25+, CD38+, CD79b+, CD81+, CD95+, CD200+, FMC7+, HLA-DR+, slambda+	*macroscopic*: colorless clear liquid; *microscopic*: small to medium-sized lymphoid infiltrate	concordance: lymphoma evidence by FC and CP	primary B-cell CNS lymphoma with intraocular lymphoma
**#3**	lymphocytes 66%, B-cells 35%, T-cells 14%, NK-cells 17%; B-cells: CD19+, CD20+, CD5-, CD10-, CD38-, CD200+, HLA-DR+, skappa+	*macroscopic*: colorless clear liquid; *microscopic*: atypical blast-like lymphoid cells	concordance: lymphoma evidence by FC and CP	isolated B-cell intraocular lymphoma
**#4**	lymphocytes 36%, B-cells 30%, T-cells 1%, NK-cells 5%; B-cells: CD19+, CD20+, CD5-, CD38+, ckappa+	*macroscopic*: colorless clear liquid; *microscopic*: no conspicuous lymphocytes	discordance: lymphoma evidence by FC; lack of lymphoma evidence by CP	primary CNS lymphoma (DLBCL) with intraocular lymphoma
**#5**	acellular punctate	*macroscopic*: slightly yellow and turbid liquid *microscopic*: some inconspicuous lymphocytes, a few hyalocytes and erythrocytes	concordance: acellular vitrous body, i.e. no lymphocytes, according to FC; no evidence of ocular manifestation of DLBCL with CNS involvement according to CP	secondary CNS lymphoma following progress of DLBCL; no findings of intraocular lymphoma according to CP and FC
***Exclusion of intraocular lymphoma***				
**#6**	lymphocytes 5%, B-cells <1%, T-cells 2%, NK-cells 2%; no aberrant immunophenotype	NA	no evidence of intraocular lymphoma by FC; CP not performed	no findings of intraocular lymphoma
**#7**	lymphocytes 71%, B-cells <1%, T-cells 49%, NK-cells 20%; no aberrant immunophenotype	NA	no evidence of intraocular lymphoma by FC; CP not performed	no findings of intraocular lymphoma

## Data Availability

The data presented in this study are available on request from the corresponding author.

## References

[1] Freeman L.N., Schachat A.P., Knox D.L., Michels R.G., Green W.R. (1987). Clinical features, laboratory investigations, and survival in ocular reticulum cell sarcoma. Ophthalmology.

[2] Reddy E.K., Bhatia P., Evans R.G. (1988). Primary orbital lymphomas. Int. J. Radiat. Oncol. Biol. Phys..

[3] Hochberg F.H., Miller D.C. (1988). Primary central nervous system lymphoma. J. Neurosurg..

[4] Grimm S.A., McCannel C.A., Omuro A.M.P., Ferreri A.J.M., Blay J.-Y., Neuwelt E.A., Siegal T., Batchelor T., Jahnke K., Shenkier T.N. (2008). Primary cns lymphoma with intraocular involvement. Int. PCNSL Collab. Group Rep..

[5] Farrall A.L., Smith J.R. (2020). Eye involvement in primary central nervous system lymphoma. Surv. Ophthalmol..

[6] Karakawa A., Taoka K., Kaburaki T., Tanaka R., Shinozaki-Ushiku A., Hayashi H., Miyagi-Maeshima A., Nishimura Y., Uekusa T., Kojima Y. (2018). Clinical features and outcomes of secondary intraocular lymphoma. Br. J. Haematol..

[7] Soussain C., Malaise D. (2021). Primary vitreoretinal lymphoma: A diagnostic and management challenge. Blood.

[8] Cunningham E.T., Miserocchi E., Smith J.R., Gonzales J.A., Zierhut M. (2021). Intraocular Lymphoma. Ocul. Immunol. Inflamm..

[9] Tang L.-J., Gu C.-L., Zhang P. (2017). Intraocular lymphoma. Int. J. Ophthalmol..

[10] AlQahtani A., Touitou V., Cassoux N., Aknin C., Merle-Beral H., Bodaghi B., LeHoang P. (2014). More than a masquerade syndrome: Atypical presentations of vitreoretinal lymphomas. Ocul. Immunol. Inflamm..

[11] Fend F., Ferreri A.J.M., Coupland S.E. (2016). How we diagnose and treat vitreoretinal lymphoma. Br. J. Haematol..

[12] Coupland S.E., Anastassiou G., Bornfeld N., Hummel M., Stein H. (2005). Primary intraocular lymphoma of t-cell type: Report of a case and review of the literature. Graefe’s Arch. Clin. Exp. Ophthalmol..

[13] Chan C.-C., Rubenstein J.L., Coupland S.E., Davis J.L., Harbour J.W., Johnston P.B., Cassoux N., Touitou V., Smith J.R., Batchelor T.T. (2011). Primary vitreoretinal lymphoma: A report from an international primary central nervous system lymphoma collaborative group symposium. Oncologist.

[14] Coupland S.E., Bechrakis N.E., Anastassiou G., Foerster A.M.H., Heiligenhaus A., Pleyer U., Hummel M., Stein H. (2003). Evaluation of vitrectomy specimens and chorioretinal biopsies in the diagnosis of primary intraocular lymphoma in patients with masquerade syndrome. Graefe’s Arch. Clin. Exp. Ophthalmol..

[15] Raja H., Salomão D.R., Viswanatha D.S., Pulido J.S. (2016). Prevalence of *Myd88* l265p mutation in histologically proven, diffuse large b-cell vitreoretinal lymphoma. Retina.

[16] Bonzheim I., Giese S., Deuter C., Süsskind D., Zierhut M., Waizel M., Szurman P., Federmann B., Schmidt J., Quintanilla-Martinez L. (2015). High frequency of *Myd88* mutations in vitreoretinal b-cell lymphoma: A valuable tool to improve diagnostic yield of vitreous aspirates. Blood.

[17] Shi H., Zhou X., Chen B., Xiao J., Li Y., Zhou X., Zhou Q., Chen K., Wang Q. (2021). Clinical relevance of the high prevalence of *Myd88* l265p mutated vitreoretinal lymphoma identified by droplet digital polymerase chain reaction. Ocul. Immunol. Inflamm..

[18] Davis J.L., Viciana A.L., Ruiz P. (1997). Diagnosis of intraocular lymphoma by flow cytometry. Am. J. Ophthalmol..

[19] Kase S., Namba K., Iwata D., Mizuuchi K., Kitaichi N., Tagawa Y., Okada-Kanno H., Matsuno Y., Ishida S. (2016). Diagnostic efficacy of cell block method for vitreoretinal lymphoma. Diagn. Pathol..

[20] Flores-Montero J., Grigore G., Fluxá R., Hernández J., Fernandez P., Almeida J., Muñoz N., Böttcher S., Sedek L., van der Velden V. (2019). Euroflow lymphoid screening tube (lst) data base for automated identification of blood lymphocyte subsets. J. Immunol. Methods.

[21] Tanaka R., Kaburaki T., Taoka K., Karakawa A., Tsuji H., Nishikawa M., Yatomi Y., Shinozaki-Ushiku A., Ushiku T., Araki F. (2021). More accurate diagnosis of vitreoretinal lymphoma using a combination of diagnostic test results: A prospective observational study. Ocul. Immunol. Inflamm..

[22] Cantu C.A., Green C.L., Cummings T.J., Liu B., Dash R.C. (2019). Flow cytometry immunophenotyping of vitreous specimens does not contribute to diagnosis of lymphoma without supporting morphologic features. Diagn. Cytopathol..

[23] Raparia K., Chang C.C., Chévez-Barrios P. (2009). Intraocular lymphoma: Diagnostic approach and immunophenotypic findings in vitrectomy specimens. Arch. Pathol. Lab. Med..

